# Transplantation of Aceh cattle ovary into the uterus of pseudopregnant local rabbits: Effect of post-transplant stress on uterine histopathology and ovarian follicle dynamics

**DOI:** 10.14202/vetworld.2023.500-508

**Published:** 2023-03-21

**Authors:** Syafruddin Syafruddin, Tongku Nizwan Siregar, Sri Wahyuni, Gholib Gholib, Ilfa Liyandara Chairunnisa Pulungan, Muchsalmina Muchsalmina

**Affiliations:** 1 Study Program of Graduate School of Mathematics and Applied Sciences, Universitas Syiah Kuala, Banda Aceh, 23111, Aceh, Indonesia; 2 Laboratory of Clinic, Faculty of Veterinary Medicine, Universitas Syiah Kuala, Banda Aceh, 23111, Aceh, Indonesia; 3 Laboratory of Reproduction, Faculty of Veterinary Medicine, Universitas Syiah Kuala, Banda Aceh, 23111, Aceh, Indonesia; 4 Laboratory of Anatomy, Faculty of Veterinary Medicine, Universitas Syiah Kuala, Banda Aceh, 23111, Aceh, Indonesia; 5 Laboratory of Physiology, Faculty of Veterinary Medicine, Universitas Syiah Kuala, Banda Aceh, 23111, Aceh, Indonesia; 6 Study Program of Veterinary Medicine, Faculty of Veterinary Medicine, Universitas Syiah Kuala, Banda Aceh, 23111, Aceh, Indonesia

**Keywords:** cortisol, ovarian, transplantation

## Abstract

**Background and Aim:**

The increase in the levels of the cortisol hormone caused by the stress conditions generated by an ovary transplantation procedure can damage the uterus of the transplant recipient as well as the transplanted ovaries. This study aimed to analyze the histopathological changes that occur in the uterine horn of pseudopregnant local rabbits (recipients), as well as the ovarian follicular integrity of the donor Aceh cattle after transplantation.

**Materials and Methods:**

After 30 days of adaptation, all rabbits were divided into three treatment groups: R1 (the group of rabbits that underwent ovarian transplantation for 3 days, n = 5), R2 (the group of rabbits that underwent ovarian transplantation for 5 days, n = 5), and R3 (the group of rabbits that underwent ovarian transplantation for 7 days, n = 5). Pseudopregnancy induction was performed using the pregnant mare’s serum gonadotropin (PMSG) and human chorionic gonadotropin (hCG) methods. The rabbits were injected with 100 IU of PMSG intramuscularly, followed by an injection of 75 IU of hCG intravenously 3 days later. Ovarian transplantation was performed on day 8 (day 0 was the day of hCG injection). The concentration of cortisol hormone metabolites was measured from fecal samples using an enzyme-linked immunosorbent assay technique. The uterus and ovaries were collected for histopathological and follicular dynamics examination after the transplantation process was completed.

**Results:**

The mean cortisol levels (ng/g) recorded before versus after the transplant in the R1, R2, and R3 groups were 146.23 ± 17.60 versus 338.84 ± 302.79, 128.97 ± 81.56 versus 174.79 ± 101.70, and 124.88 ± 43.61 versus 321.91 ± 221.63 (p < 0.05), respectively. The examination of the histopathological appearance of the uterus revealed edema in the uterine lumen, hyperemia and hemorrhage in the endometrium, necrosis of the epithelium, and infiltration of inflammatory cells. Hemorrhage and hyperemia were severe and filled the endometrium in the R1 compared with the R2 and R3 animals. Ovarian follicle development occurred in all treatment groups, although some histopathological features were observed. The number of tertiary follicles in R1, R2, and R3 animals was 24.67 ± 7.37, 20.67 ± 7.57, and 9.67 ± 3.79 (p < 0.05), respectively.

**Conclusion:**

Based on the results of this study, it can be concluded that the transplantation of ovaries from Aceh cattle into pseudopregnant local rabbits triggered an increase in the levels of the cortisol hormone and uterine histological changes; however, follicles were still detected at various stages of development in the transplanted Aceh cattle ovaries. The results of this study are valuable for clinicians and researchers because they provide information regarding an alternative *in vivo* ovarian preservation technique using pseudopregnant rabbits.

## Introduction

Aceh cattle are one of the original local cattle families that are protected by the Indonesian government. Aceh cattle are a wealth of genetic resources, but are currently threatened, both in terms of quantity and purity, due to the uncontrolled entry of foreign cattle breeds into the country [1]. Some measures have been adopted to increase the population and productivity of Aceh cattle through the application of various reproductive technologies, including estrus synchronization technology using prostaglandin F2 alpha [2, 3], estrus synchronization with presynch–ovsynch [4], the application of artificial insemination (AI) on Aceh cattle using superior cow breeds [5], and superovulation using pregnant mare’s serum gonadotropin (PMSG) and human chorionic gonadotropin (hCG) [6].

The implementation of embryo transfer (ET) begins by obtaining good-quality embryos that are transferrable to the recipients. Embryos stem from an ovum that has undergone fertilization as a result of the mating process, either naturally or through AI. However, it is generally difficult to obtain good-quality embryos. One approach that can be used to overcome this obstacle is the production of embryos using *in vitro* fertilization (IVF) technology [7]. The ovarian follicles used in IVF technology can be obtained from cow ovaries as slaughterhouse wastes, which are disposed of after the cow is slaughtered. The ovaries can be used as a source of follicles that are ready to be fertilized. However, the ovaries are short-lived, even when stored at low temperature. As a consequence, ovarian preservation technology is needed to maintain the internal quality of the follicles. Interspecies implantation with or without vitrification of the ovarian cortex or the entire ovary as one the most popular technology for ovarian preservation [8]. Ovarian transplantation can be achieved using experimental animals, such as rabbits. Rabbits are an animal model that is commonly used in studies of reproductive endocrinology [9], detection of hormone receptors [10], and ovarian transplantation, as a source of oocytes [11]. Ovarian transplantation can be performed on pseudopregnant rabbits because, at the beginning of pregnancy, the glands found in the uterine endometrium of rabbits actively produce and stimulate secretions into the uterine lumen, as a nutritional reserve for the embryo before implantation. This energy source can be utilized to maintain and slow down the autolysis process in the transplanted organs [12].

One of the effects of transplantation or other types of surgery on animal models is the emergence of stress conditions [13], which may cause rejection of the transplanted organ by the host’s immune system. This can affect the success rate of transplantation [14]. The disadvantage of using rabbits for organ transplantation is that these animals are vulnerable to the stress resulting from the treatment. Although rabbits are popular animal models, they are very susceptible to stress [15]. Jang *et al*. [16] found an increase in cortisol after surgery in rabbits with gastrointestinal disturbance. Stress caused by transplantation can stimulate the secretion of corticotropin-releasing hormone (CRH), which triggers the pituitary gland to release adrenocorticotropic hormone (ACTH) and increase the level of cortisol. Moreover, CRH plays an important role in the regulation of follicular development [17]. Wu *et al*. [18] reported that cortisol can upregulate 11b-HSD1 in the granulosa cells of patients with polycystic ovary syndrome. In turn, an increase in cortisol levels will cause metabolic disturbances in the ovaries. Stress can also cause damage to the uterus of pseudopregnant rabbits. This stress is attributable to surgery and anesthesia [19]. In addition, surgical incisions can also lead to stress. Therefore, in the early period of transplantation, it is assumed that the stress level is increased; therefore, the uterine damage at the beginning of the transplantation process may be greater than that occurring in the subsequent period. In addition, the increase in the pseudopregnancy time will increase secretion from the endometrial glands. This secretion functions as a micronutrient during early pregnancy [20]. Furthermore, the increase in secretion will reduce the chance of adhesion between the ovaries and the uterus, thus minimizing the damage.

Therefore, this study aimed to analyze the histopathological changes that occur in the uterus of local rabbits (recipients), as well as the integrity of the ovarian follicle of Aceh cattle after transplantation.

## Materials and Methods

### Ethical approval

This study obtained an ethical certificate from the Ethics Committee of the Faculty of Veterinary Medicine, Syiah Kuala University (No. 139/KEPH/VII/2020).

### Study period and location

The study was conducted from January to March 2022 at the Experimental Animals Unit, Laboratory of Clinic (Surgery), Laboratory of Physiology, and Laboratory of Pathology, Faculty of Veterinary Medicine, Universitas Syiah Kuala, Banda Aceh.

### Research design

Fifteen local rabbits aged 18–24 months with body weights of 1.5–2.9 kg were used as recipients for ovarian transplantation from Aceh cattle and divided into three treatment groups, namely, R1, R2, and R3, with each group including five rabbits. All rabbits were adapted for 30 days at the UPT Experimental Animals Faculty of Veterinary Medicine, Universitas Syiah Kuala Banda Aceh. Furthermore, all rabbits were maintained in separate cages measuring 60 cm × 43 cm × 48 cm. During maintenance, the rabbits were given pellets twice a day, whereas drinking water was provided *ad libitum*.

After the adaptation period, pseudopregnancy was induced in the rabbits using the PMSG and hCG methods. These methods were chosen based on the report by Syafruddin *et al*. [21], which state that pseudopregnancy induction using PMSG and hCG yields better results than methods that use hCG, gonadotropin hormone-releasing hormone, or artificial copulation. Rabbits with pseudopregnancy induced using PMSG and hCG exhibited a similar level of progesterone as did normal pregnant rabbits until the 8^th^ day, that is, 79.67 ± 26.1 and 61.73 ± 20.7 ng/mL, respectively, as well as a similar number and size of corpora lutea. The number and diameter of follicles and corpora lutea in rabbits with pseudopregnancy induced by PMSG and hCG versus normal pregnant rabbits on day 8 were 8.8 ± 1.90 versus 6.87 ± 1.58 and 1.65 ± 0.37 versus 1.39 ± 1.17 mm, respectively. Briefly, the rabbits were injected with 100 IU o PMSG (PG 600, Folligon™, Intervet, Boxmmer, and Holland) intramuscularly, followed by an intravenous injection of 75 IU of hCG (Chorulon, Intervet, Boxmeer, and Holland) (R3) 3 days later [22]. On day 8 (day 0 was the day of hCG injection), small pieces of ovaries from Aceh cattle were transplanted into the uterus of pseudopregnant rabbits. The storage times of cattle ovaries after transplantation into the uterus of rabbits R1, R2, and R3 were 3, 5, and 7 days, respectively.

### Surgical procedure

Female rabbits that had undergone pseudopregnancy induction were subjected to general anesthesia by injecting 0.1 mL/kg of body weight of tiletamine–zolazepam (Zoletil, Virbac, and France) intramuscularly. Surgery in the rabbits, which was carried out under sterile conditions, began by making a 3–4 cm incision on the linea alba of the skin with the animal in a dorsal recumbency position, to allow reaching the uterus. An incision was then made in the dorsal part of the uterine horns, to insert the pieces of the ovary from Aceh cattle.

After the transplantation procedure, the incision in the endometrium was sutured with a continuous simple suture pattern, whereas the perimetrium was sutured using a Lambert continuous suture. Subsequently, the peritoneum was also sutured using a continuous simple suture pattern, as was the transversus abdominis muscle, followed by the suturing of the skin using a simple interrupted suture pattern. To suture the uterus, absorbable threads were employed (RTMED PGA 2/0, Shandong Haidike Medical Products Co. Ltd., China). Furthermore, to suture the skin area, peritoneum, and transversus abdominis muscle, non-absorbable threads were applied (OneMed 3/0, PT. Jayamas Medica Industri, Sidoarjo, Indonesia). After the transplantation process was completed, the incision wound was monitored and managed by administering gentamicin (Genoint, Erela, Semarang, Indonesia) twice daily [11].

### Fecal sample collection and extraction

Fecal samples were collected from all rabbits 3 days before transplantation and up until the ovaries were removed from the uterus. A fecal sample was collected immediately after defecation, and 5–10 g was placed in a sample tube and then stored in a freezer at –20°C before extraction. Fecal samples were extracted using a method described by Gholib *et al*. [23]. Before the extraction process, all frozen fecal samples were thawed at 50°C for 1–2 h. Then, the samples were homogenized and ~0.55 g of the fresh fecal samples was placed in tubes containing 4.5 mL of 80% methanol. The fecal samples were then extracted using a multivortexer (Brand, USA) at a speed of 123× *g* for 10 min. Subsequently, the samples were centrifuged at 1107× *g*) for 10 min. Finally, the supernatant was collected, placed in microtubes, and stored in a freezer at −20°C before the measurement of the concentration of cortisol metabolites.

### Cortisol hormone measurements

The procedure for measuring the concentration of cortisol metabolites in the fecal extract was performed according to Gholib *et al*. [24] and Gholib *et al*. [25]. A non-invasive method of measurement of cortisol levels in rabbit fecal samples was used, to minimize stress in the rabbits. Therefore, blood sample collection (which is an invasive method) was not performed in this work. The extracted sample was diluted with an assay buffer solution. A total of 50 μL of the diluted extract, standards, and quality controls were assayed on a microplate. Then, 50 μL of the antibody and 50 μL of the enzyme conjugate were added to each well and mixed. The mixture was incubated overnight at 4°C–8°C. After the incubation, the plate was washed 4 times with a phosphate-buffered saline washing solution (pH 7.4) containing 0.05% Tween 20 and then blotted dry. Streptavidin–peroxidase (S-5512, Sigma, Germany) in assay buffer (150 μL) was added to each well and the plate was incubated at room temperature (RT) for 30 min in the dark. Following incubation, the plate was washed again for four times, and 150 ml substrate solution consisting of 1.2 mM H_2_O_2_ and 0.4 mM 3,3’,5.5’-tetramethylbenzidine was added to each well. Furthermore, the plate was incubated again at RT in the dark for 30–45 min, depending on the color change. The enzyme reaction was then stopped by adding 50 μl of 2M H_2_SO_4_ to each well of the plate. Absorbance in each well of the plate was measured using an enzyme-linked immunosorbent assay reader (xMark™ Microplate Absorbance Spectrophotometer, Bio-Rad Laboratories Inc., California, USA) at 450 nm.

### Collection of Aceh cattle ovaries from the uteruses of rabbits

The ovaries of Aceh cattle were removed from the uteruses o rabbits through surgery according to the treatment group. The ovaries were then fixed with 10% neutral-buffered formalin, followed by immersion in a series of 70% to absolute ethanol, clearance in xylene, infiltration in liquid paraffin, and embedding in paraffin (to form blocks). Subsequently, a paraffin block containing ovarian samples was cut into a 3 μm thickness using a rotary microtome. The tissue sections were placed on the surface of glass slides and stained with Hematoxylin and Eosin solution. This procedure was carried out according to Kiernan [26].

Observations of the histopathology of the rabbit uterus and the morphology and number of ovarian follicles in Aceh cattle were carried out using a light microscope (Olympus CX31, Japan). The histopathological observation of the uterine horns of rabbits revealed the presence of erosion and hyperplasia of the endometrial columnar epithelium and uterine gland atrophy, edema, hemorrhage, and infiltration of inflammatory cells, such as neutrophils, lymphocytes, and macrophages. Subsequently, the types of ovarian follicles (primordial, primary, secondary, and tertiary follicles) were examined. The number of follicles was calculated at various stages of folliculogenesis. Primordial follicles contain small oocytes with a single layer of flattened granulosa cells, but no zona pellucida [27]. Primary follicles are characterized by an oocyte diameter of >20 μm, with the oocyte being surrounded by a single layer of cuboidal epithelial cells (granulosa cells) and the zona pellucida. In turn, secondary follicles are characterized by an oocyte diameter of 70 μm, with the oocyte being surrounded by several layers of granulosa cells, the zona pellucida, and the antrum, which begin to spread over the granulosa layer. Finally, tertiary follicles are characterized by an oocyte diameter of 70 μm, with the oocyte being surrounded by several layers of granulosa cells, the zona pellucida, and a scattered antrum, which develops into a single antrum [28].

### Statistical analysis

Data pertaining to cortisol metabolite concentration were analyzed using a two-way analysis of variance, whereas the number of follicles at various stages of development was analyzed using a one-way analysis of variance. Data analyses were carried out using the Statistical Package for the Social Sciences (SPSS) program, version 21 (IBM SPSS Statistics for Windows, Version 21.0, IBM Corp., USA). Furthermore, the histopathological description of the rabbit uterus and Aceh cattle ovaries was presented descriptively.

## Results

The mean cortisol hormone concentration in pseudopregnant local rabbits before the transplantation (H-3) of ovaries in the R1, R2, and R3 groups was 146.23 ± 17.60, 128.97 ± 81.56, and 124.88 ± 43.61 ng/g (p > 0.05), respectively, as presented in Table-1. The cortisol levels in all groups before transplantation showed no significant differences (p > 0.05). In contrast, after transplantation, the cortisol levels increased significantly in the R1 and R3 groups (p < 0.05), but not in the R2 group (p > 0.05). The comparison of the stress levels of the rabbits and the length of the ovaries in the uterus for 3 days (R1) revealed that the rabbits had high-stress levels, which were significantly different (p < 0.05) between R2 (5 days) and R3 (7 days) animals.

**Table-1:** Metabolite cortisol concentration (ng/g) of pseudopregnant local rabbits before and after transplant.

Group	Group and duration of ovarian in the uterus of rabbits		

	R1 (3 days)	R2 (5 days)	R3 (7 days)
Before transplant	146.23±17.60^aA^	128.97±81.56^aA^	124.88±43.61^aA^
After transplant	338.84±302.79^bB^	174.79±101.70^aA^	321.91±221.63^bB^

^a,b^Different superscripts on the same line show significant differences (p < 0.05). ^A,B^Different superscripts in the same column show significant differences (p < 0.05)

**Figure-1:**
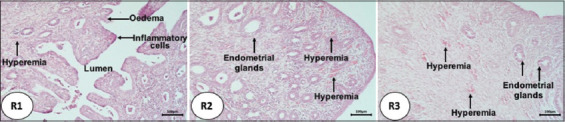
Histopathological changes of endometrial glands of rabbits undergoing ovarian transplantation. R1: transplant for 3 days; R2: transplant for 5 days; R3: transplant for 7 days, Hematoxylin and Eosin staining, magnification 40×.

**Figure-2:**
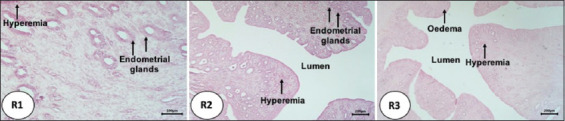
Hyperemia, edema, and inflammatory cell infiltration of inflammatory cells in the uterus of rabbits undergoing ovarian transplantation. R1: transplant for 3 days; R2: transplant for 5 days; R3: transplant for 7 days, Hematoxylin and Eosin staining, magnification 100×.

**Figure-3:**
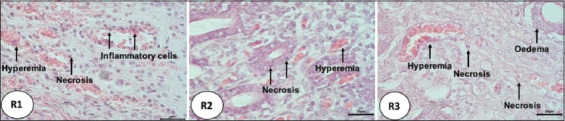
Hyperemia and necrosis in the uterus of rabbits undergoing ovarian transplantation. R1: transplant for 3 days; R2: transplant for 5 days; R3: transplant for 3 days, Hematoxylin and Eosin staining, magnification 100×.

In both R2 and R3 rabbits, the results of microscopic observations showed an increase in the size of the uterine glands (Figure-1), with the greatest increase in size observed in R2 animals, followed by a decrease in R3 rabbits. The number of endometrial glands was greater in the R2 and R3 groups compared with the R1 group. After the transplantation of the ovaries, the rabbit uteruses exhibited changes that were characterized by the presence of hyperemia, hemorrhage, edema, necrosis, and infiltration of inflammatory cells (Figure-2). The R1 rabbits exhibited edema in the uterine lumen, endometrial hyperemia, and inflammatory cell infiltration. In addition, the hemorrhage and hyperemia were severe and filled the endometrium. In turn, R2 animals (with ovarian transplantation lasting for 5 days) exhibited hyperemia of the endometrium and necrosis of the endometrial epithelium (Figure-3). The hyperemia that occurred in R2 and R3 animals was not as severe as that observed in R1 rabbits. Although several histopathological parameters were observed in the uterine horns of pseudopregnant rabbits after ovary transplantation, identification of cytokine types present in the ovarian follicular fluid was not carried out; rather, the histopathological examination performed in this study was intended to determine the changes in uterine horn tissue and the possibility of inflammation after surgery for ovary transplantation.

**Figure-4:**
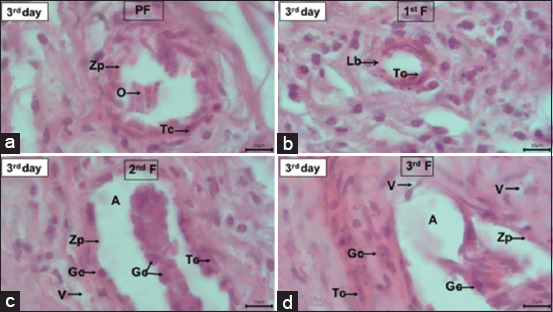
Ovarian follicles of Aceh cattle after being transplanted into the uterus of a local rabbit, pseudopregnant on the 3^rd^ day. (a) Primordial follicle (PF), (b) primary follicle (1^st^P), (c) secondary follicle (2^nd^F), and (d) tertiary follicle (3^rd^T) underwent tissue structure changes. The oocyte (O) was only present in FP; Theca (Tc) and granulosa (Gc) cell layers were not intact and separated from the basal lamina (Lb); Gc was separated from the layer and entered the follicular antrum (A); zona pellucida (Zp) in 1^st^F and 3^rd^F was not intact, and there was a vacuole (V) in the Tc layer. Hematoxylin and Eosin, magnification 1000×.

**Figure-5:**
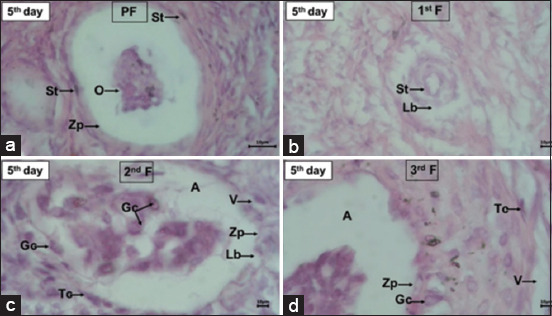
Ovarian follicles of Aceh cattle after being transplanted into the uterus of New Zealand White rabbits pseudopregnant on day 5. (a) Primordial follicle (PF), (b) primary follicle (1^st^ F), (c) secondary follicle (2^nd^ F), and (d) tertiary follicle (3^rd^ F) which have undergone changes in tissue structure. Oocyte (O) still observed in PF. Theca cells (Tc) and granulose cells (Gc) layers were not intact and separated from the lamina basalis (Lb), Gc was separated from the layers and enters to the follicular antrum (A), zona pellucida (Zp) in 2^nd^ F and 3^rd^ F were not intact, and there was vacuoles (V) in the Tc layers. Hematoxylin and Eosin staining, magnification 1000×.

**Figure-6:**
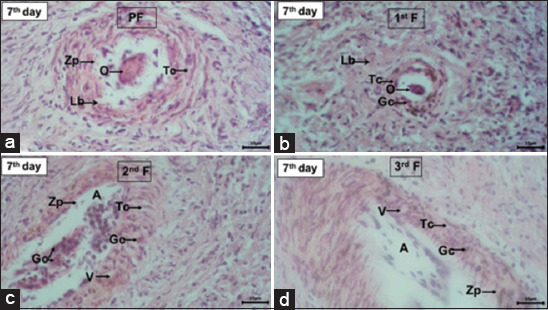
Ovarian follicles of Aceh cattle after being transplanted into the uterus of New Zealand White rabbits pseudopregnant on day 7. (a) Primordial follicle (PF), (b) primary follicle (1^st^ F), (c) secondary follicle (2^nd^ F), and (d) tertiary follicle (3^rd^ F) which have undergone changes in tissue structure. Oocyte (O) still observed in PF. Theca cells (Tc) and granulose cells (Gc) layers were not intact and separated from the lamina basalis (Lb), Gc was separated from the layers and enters to the follicular antrum (A), zona pellucida (Zp) in 2^nd^ F and 3^rd^ F were not intact, and there was vacuoles (V) in the Tc layers. Hematoxylin and Eosin staining, magnification 1000×.

The results of the histological observations of the transplanted ovaries revealed the presence of follicles at different stages, that is, primordial, primary, secondary, and tertiary follicles (Table-2), although with some damage. The damage to ovarian follicles in R1, R2, and R3 animals is presented in Figures-4–6. The damage to the ovarian follicles included changes in the tissue structure, disruption of the theca cell layer and granulosa cell, separation of granulosa cells from the layer and their entry into the antrum, an incomplete zona pellucida, and the formation of many vacuoles between the granulosa and theca cell layers. These observations also revealed intact oocytes in the primary follicles (R1 and R2) and in the primordial and primary follicles (R3).

**Table-2:** The average number of ovarian follicles (±standard deviation) of Aceh cattle after transplant into uterine of pseudopregnant local rabbits.

Post-transplant (days)	Average number of follicles			

	Primordial follicle	Primary follicle	Secondary follicle	Tertiary follicle
3	106.0±12.0	85.0±27.0^ab^	35.0±7.0	24.67±7.37^b^
5	54.33±13.05	38.67±12.10^a^	35.33±13.01	20.67±7.57^b^
7	95.67±37.42	104.33±28.01^b^	63.67±27.61	9.67±3.79^a^

^a,b^Different superscripts on the same line show significant differences (p < 0.05)

## Discussion

This study provided data on the preservation of ovarian tissues in Aceh cattle using interspecies transplantation techniques into the uterine horn of local pseudopregnant rabbits. Data regarding post-transplantation effects on cortisol levels and histopathological changes in the uterine horns of local rabbits as transplant recipients were reported. Additionally, ovarian follicular integrity observed until day 7 after transplantation was also presented in this study. The use of pseudopregnant local rabbits as ovarian transplant recipients in this study was appropriate, as Sumarmin [11] reported previously that pseudopregnant rabbits can be used as a source of oocytes for ovarian transplantation. Despite the use of rabbits in various studies, such as those of reproductive endocrinology [9] and hormone receptors [10], in the present study, we focused on using rabbits for interspecies ovarian transplantation. The results of the ovarian transplantation performed here revealed the presence of several follicles that contained intact oocytes. This indicates that the lumen and endometrial lining of the pseudopregnant rabbits can maintain ovarian tissues for a certain period.

The results pertaining to the mean cortisol concentration before the transplantation procedure showed that all rabbits had the same stress level (p > 0.05). However, these results do not indicate that the degree of stress in these rabbits is related to the method of examining cortisol through stool, as well as the limited data available regarding the normal cortisol levels in local rabbits. Jang *et al*. [16] revealed that the normal cortisol concentration in New Zealand rabbits before surgery was 1.32 ± 0.25 μg/dL. This observation suggests that the adaptation period was effective and the rabbits were in the same comfort condition.

The increase in cortisol concentration after the transplantation procedure detected in this study was consistent with the results of our initial study. The mean cortisol metabolite concentration detected in rabbits 3 days before and after the transplantation of Aceh cattle ovaries into the uteruses of pseudopregnant rabbits was 125.12 ± 74.68 and 433.94 ± 207.44 ng/g, respectively [29]. The increase in cortisol levels observed after transplantation (R1 and R3 groups) in this study may be associated with surgery and anesthesia. The surgery increases pituitary hormone secretion and activates the sympathetic nervous system in response to the stress caused by it [30]. Changes in anterior pituitary secretion have the secondary effect of increasing hormone secretion in target organs [19]. The cortisol levels will increase rapidly after surgery as a result of the stimulation of ACTH. Surgery is the main activator that can increase ACTH and cortisol secretion, which is also affected by the duration of the surgical procedure [31]. In addition, anesthesia may play a role in elevating the stress levels of the experimental animals. In this study, general anesthesia was used. The response to the stress caused by general anesthesia followed by surgery has been documented in rabbits [32, 33]. Anesthesia and surgery have been reported to produce hormonal and metabolic changes that trigger anterior pituitary hormone release. However, other reports have stated that general anesthesia causes a lower stress index than local anesthesia [34]. In this study, we did not use pre-emptive analgesia before the surgical procedure because the procedure used here refers to a previous study conducted by Sumarmin [11], in which pre-emptive analgesia was not administered before surgery.

The decrease in stress levels observed after transplantation in R2 and R3 animals compared with R1 animals may be related to increased uterine gland secretion. Increased secretion of the uterine glands will reduce the mechanical trauma between the ovaries and the uterus. Burton *et al*. [20] suggested that the endometrial glands serve as an important pathway for micronutrient transfer during early gestation. The increase in uterine gland secretion is associated with increased number and size of uterine glands. The size of the uterine glands reached a maximum at R2 and was then decreased at R3. The reduction in the size of the uterine glands at R3 was also accompanied by escalated cortisol levels, which reinforces the assumption that post-transplant stress is primarily caused by mechanical trauma due to adhesions between the ovaries and the uterus. In turn, mechanical trauma can cause necrosis of the lamina epithelium of the endometrial mucosa. Trauma can be accompanied by hematological changes due to the stress response after surgery [35].

The number of endometrial glands also tended to be higher in R2 and R3 compared with R1 animals. These results are in accordance with the statement of Amira *et al*. [36], that is, the number and size of endometrial glands increased during pregnancy in rats and were controlled by progesterone. Syafruddin *et al*. [21] proved that the concentration of progesterone on day 6 after the induction of pseudopregnancy in local rabbits with PMSG and hCG differed significantly from that observed on day 2, with concentrations of 6.39 ± 4.65 and 3.54 ± 3.82 ng/mL, respectively. This increase seems to be related to an increase in the number of endometrial glands. The number and size of endometrial glands in R2 rabbits were greater than those observed in R3 animals. This is probably attributable to the fact that the pseudopregnancy period in the R3 animals was almost over. Pseudopregnancy in rabbits generally lasts for 16–18 days. As the transplant was carried out on day 8 of pseudopregnancy, followed by 7 days of transplantation, it can be assumed that the pseudopregnancy period would end, followed by a decrease in the concentration of progesterone, which controlled the number and size of the endometrial glands of the rabbits.

After transplantation, in R1 animals, inflammatory cells accumulated at various sites due to inflammation, likely caused by the rejection of the ovaries of the Aceh cattle by the uteruses of rabbits. Inflammation is a response to tissue injury and infection in body cells. The process of inflammation triggers a vascular reaction, that is, blood elements, including leukocytes, concentrate at the site of injury or infection. This process is a form of body protection aimed at neutralizing and eradicating harmful agents, such as microorganisms, thus enabling the tissue to return to a normal state and work properly [37]. The hyperemia observed here appeared to result from the entry of a foreign object into the uterus of the pseudopregnant rabbits. The ovary, as a foreign body, “infected” the uterus of the pseudopregnant rabbits, resulting in severe hyperemia.

The hyperemia detected in R2 and R3 animals was less severe than that observed in R1 rabbits. This is probably attributable to nutritional assistance from the endometrial glands, which were higher than those observed in the R2 and R3 groups. The necrosis that occurred in R2 animals was also less severe than that detected in R1 rabbits. In the R3 group, the endometrium appeared more normal and exhibited less damage; the only damage observed was necrosis and mild hyperemia.

Necrosis of the laminae epithelium of the endometrial mucosa can be triggered by several factors, such as temperature, radioactive light, lack of blood supply, toxins, and mechanical trauma [35]. In this study, necrosis was potentially caused by mechanical trauma and individual injuries triggered by friction or adhesion between the ovary and the uterine wall, or the stress caused by the rejection of the implanted tissue. The results of this study showed that there was an increase in cortisol concentration after ovarian transplantation into pseudopregnant local rabbits. The mean cortisol levels (ng/g) recorded before versus after transplantation on days 3, 5, and 7 were 146.23 ± 17.60 versus 338.84 ± 302.79; 128.97 ± 81.56 versus 174.79 ± 101.70; and 124.88 ± 43.61 versus 321.91 ± 221.63, respectively.

In this study, the transplant procedure was considered to be successful because some follicles remained in the various stages of development. This is in accordance with the report of Sumarmin *et al*. [38], who stated that the success of the transplantation can be proved by the discovery of follicles; in fact, the presence of follicles proves that the epithelial structure that protects the ovarian cortex is intact, thus ensuring that metabolism can occur normally. In several histological images (Figures-5 and 6) of the transplanted Aceh cattle ovaries, several artifacts on the tissue surfaces were observed. These artifacts may have been formed due to various factors, such as the ovarian transplantation process in the uterus, which causes damage to the tissue surface. Other parameters were related to technical factors, such as sample preparation, tissue fixation, and embedding.

There was a decrease in the number of primordial follicles on the 5^th^ and 7^th^ days compared with the 3^rd^ day after transplantation, although these differences were not significant (p > 0.05). Sumarmin *et al*. [38] also reported that the average number of sheep primordial follicles decreased with increasing transplantation time. The number of primary and secondary follicles increased in accordance with the increase in the number of days of transplantation, although only secondary follicles exhibited a significant difference (p < 0.05). This demonstrates that the ovarian epithelial tissue was still good and that the cortex and medulla of the ovary had not degenerated that time point. The average number of tertiary follicles decreased compared with that observed for the primordial, primary, and secondary follicles.

On day 7 post-transplantation, the number of tertiary follicles was significantly decreased (p < 0.05). This was probably caused by the apoptotic process induced in the follicles as a result of stress stimulation [39]. Table-2 shows that there was an increase in cortisol concentration as a result of transplantation. Increased cortisol will trigger oxidative stress and induce the apoptosis of granulosa cells, which in turn results in disturbances in folliculogenesis, thereby disrupting the communication between granulosa cells and oocytes. This disruption results in an impaired nutrient supply and oocyte maturation [40].

The damage to the follicular tissue observed in this study was assumed to be related to increased stress in rabbits, leading to the accumulation of reactive oxygen species (ROS) in the ovaries. The oxidative stress arising from the accumulation of ROS stimulates the apoptosis of granulosa cells and oocytes, which decreases oocyte quality [41]. The same damage was also reported in mice with stress induced by several different stressors. The stress response in ovarian histology is characterized by cellular degeneration, inflammatory cell infiltration, and follicle attrition [42]. In this study, we did not identify the inflammatory cytokines in the follicular fluid. Inflammation caused by ovarian surgery and transplantation in the host can occur and can be demonstrated by cytokine assays.

The research reported here was limited to the preservation of the ovaries of the Aceh cattle through ovarian transplantation techniques and has not been achieved using the ET procedure. To reduce stress in this study, we applied non-invasive methods for the measurement of the cortisol hormone using a rabbit fecal sample. However, we did not administer pre-emptive analgesia before surgery to reduce stress. This research consisted in a preliminary study that warrants follow-up by several studies, to support the initial data obtained here. Moreover, further research is needed to develop ovarian preservation techniques with the ultimate goal of maintaining oocyte viability in the context of IVF, especially in Aceh cattle.

## Conclusion

This study had several limitations that can be improved in further studies. The stress response in local rabbits, as recipients of ovarian transplantation in this study, can be reduced through the administration of pre-emptive analgesia or antidepressant medication. Furthermore, several recent studies have demonstrated that the use of intact ovaries yields a better number of follicles. In contrast, we used the ovaries of Aceh cattle, which had been divided into small pieces and adjusted to the size of the uterine lumen of the local rabbit. In future studies, it is necessary to carry out additional procedures, such as administering antidepressant drugs before surgery and using intact ovaries by transplanting rabbits with different breeds that have larger uterine horns so as to obtain better results. The study can also be continued by evaluating the quality of the oocytes in the transplanted ovarian follicles and followed by *in vitro* fertilization procedures.

## Authors’ Contributions

SS, TNS, and SW: Designed the study, sample collection and processing, interpreted of the data and major contributor in writing of the manuscript. SW: Performed the study and edited the manuscript. ILCP and MM: Assisted the sample collection and histological preparation. GG: Performed hormone and data analysis. All authors have read, reviewed, and approved the final manuscript.
